# Genetic polymorphisms of the dopamine and serotonin systems modulate the neurophysiological response to feedback and risk taking in healthy humans

**DOI:** 10.3758/s13415-012-0108-8

**Published:** 2012-07-19

**Authors:** I. Heitland, R. S. Oosting, J. M. P. Baas, S. A. A. Massar, J. L. Kenemans, K. B. E. Böcker

**Affiliations:** 1 Department of Experimental Psychology & Psychopharmacology, Utrecht University, Heidelberglaan 2, 3508 TC Utrecht, The Netherlands; 2 Division of Psychopharmacology, Utrecht Institute of Pharmaceutical Sciences, Utrecht, The Netherlands; 3 Alan Turing Institute Almere, Almere, The Netherlands; 4 Helmholtz Research Institute, Utrecht, The Netherlands; 5 Cognitive Neuroscience Laboratory, Duke-NUS Graduate Medical School Singapore, Singapore, 169857 Singapore

**Keywords:** Dopamine, Serotonin, EEG, Feedback, Reward, Risk taking, DAT, 5HTTLPR, COMT

## Abstract

Genetic differences in the dopamine and serotonin systems have been suggested as potential factors underlying interindividual variability in risk taking and in brain activation during the processing of feedback. Here, we studied the effects of dopaminergic (dopamine transporter [DAT1], catecholamine-O-methyltransferase val158met [COMT]) and serotonergic (serotonin transporter [5HTTLPR]) polymorphisms on risk taking and brain responses following feedback in 60 healthy female subjects. The subjects completed a well-established experimental gambling paradigm while an electroencephalogram was recorded. During the task, risk-taking behavior and prefrontal brain responses (feedback-related negativity [FRN]) following monetary gains and losses were assessed. FRN amplitudes were enhanced for nine-repeat-allele carriers of the DAT1 and short-allele carriers of 5HTTLPR, which are both presumably linked to less transporter activity and higher neurotransmitter levels. Moreover, nine-repeat DAT1 carriers displayed a trend toward increased risk taking in general, whereas 5HTTLPR short-allele carriers showed decreased risk taking following gains. COMT val158met genotype was unrelated to FRN amplitude and average risk taking. However, COMT met/met carriers showed a pronounced feedback P3 amplitude independent of valence, and a gradual increase in risk taking during the gambling task. In sum, the present findings underline the importance of genetic variability in the dopamine and serotonin systems regarding the neurophysiology of feedback processing.

The neural mechanisms underlying action outcome evaluation are essential for flexible adaptation of behavior, and they are related to interindividual variability in risk-taking behavior. Part of this variance has been attributed to differences within the dopamine (DA) and the serotonin (5HT) systems (Jocham & Ullsperger, 2009). Holroyd and Coles (2002) put forward an influential theory regarding the neuroanatomical and neurochemical locus of performance monitoring and feedback processing. Their account is based on studies in primates (Montague, Hyman, & Cohen, 2004; Schultz, 2002) that showed an increase in mesencephalic DA firing if an outcome is better than expected (such as a correct response or reward) and ceasing of DA neuron firing if an outcome is worse than expected (such as an error or nonreward). According to Holroyd and Coles, such reward prediction errors are conveyed by the human midbrain DA system. Moreover, they argued that these transient DA dips are projected to frontal cortical areas, among them the anterior cingulate cortex (ACC). In the ACC, these prediction error signals disinhibit cortical pyramidal neurons, which in turn serve to adapt subsequent behavior.

Electrophysiological studies in humans have shown that the ACC activity caused by this prediction error manifests itself as a negative deflection in activity peaking at frontocentral scalp sites. This negativity was found after an erroneous response was made and was termed the “error-related negativity” (ERN; Gehring, Goss, Coles, Meyer, & Donchin, 1993). It has also been found after external feedback was given that signaled an error or nonreward (feedback-related negativity, or FRN; Gehring et al., 1993; Gehring & Willoughby, 2002; Nieuwenhuis, Holroyd, Mol, & Coles, 2004; Potts, Martin, Kamp, & Donchin, 2011). Studies on the FRN have been consistent in showing that its magnitude is sensitive to both negative feedback (Gehring & Willoughby, 2002; Hajcak, Moser, Holroyd, & Simons, 2006; Holroyd & Coles, 2002; Holroyd, Hajcak, & Larsen, 2006) and positive feedback (Holroyd & Krigolson, 2007). It is thought to reflect a learning signal, promoting the avoidance of behaviors that lead to bad outcomes (Holroyd & Coles, 2002; Nieuwenhuis et al., 2004). Source localization using electroencephalography (Gehring & Willoughby, 2002; Segalowitz et al., 2010) and functional magnetic resonance imaging (Holroyd et al., 2004; Knutson, Westdorp, Kaiser, & Hommer, 2000) has confirmed the ACC as a possible generator of this prediction error.

Besides the aforementioned FRN, which is sensitive to valence, previous research has identified a second valence-unspecific event-related potential (ERP) correlate of feedback processing called the “feedback-related positivity” or “feedback P3,” which peaks approximately 300–400 ms following feedback and is sensitive to reward magnitude (Kamarajan et al., 2009; Yeung & Sanfey, 2004). Further evidence showing a dissociation between the FRN and feedback P3 originates from a recent study (Chase, Swainson, Durham, Benham, & Cools, 2011) demonstrating that the FRN reflects prediction error and associated reinforcement-learning-based adjustment of decision values, whereas the feedback P3 reflects adjustment of behavior based on explicit rules.

Although the involvement of dopamine in feedback processing is widely agreed on, its precise anatomical and temporal locus is still unknown. Moreover, surprisingly few genetic association studies have been conducted to validate the assumptions of the dopaminergic locus of feedback processing (Jocham & Ullsperger, 2009; Ullsperger, 2010). Following reinforcement learning theory (Holroyd & Coles, 2002), it would be expected that prefrontal DA activity would have an impact on both the FRN and ERN. This hypothesis has been addressed in several studies (Frank, D’Lauro, & Curran, 2007; Krämer et al., 2007; Marco-Pallarés et al., 2009) that investigated the val158met polymorphism in the catechol-O-methyltransferase (COMT) gene as an index of prefrontal DA activity. COMT is an enzyme that degrades dopamine (as well as other catecholamines) and serves as the main mechanism of DA clearance in the prefrontal cortex, with little effect on striatal DA reuptake (Egan et al., 2001; Gogos et al., 1998; Meyer-Lindenberg et al., 2005). It should be noted, however, that prefrontal DA interacts with DA transmission in the striatum (see, e.g., Bilder et al., 2004), making it likely that altered prefrontal DA levels lead to corresponding changes of DA transmission in the basal ganglia. Corresponding to the mainly prefrontal locus of COMT, Marco-Pallarés et al. (2009) reported an effect of the COMT val158met polymorphism on FRN amplitude. In contrast, ERN amplitude was found to be independent of genetic variability in COMT (Frank et al., 2007; Krämer et al., 2007). On the basis of the absence of an effect of prefrontal DA on the ERN, the latter authors proposed that striatal DA systems and genetics might be more involved than prefrontal DA in error processing. Converging evidence for striatal involvement on the generation of the ERN has been provided by studies investigating Parkinson’s disease (e.g., Falkenstein et al., 2001; Holroyd, Praamstra, Plat, & Coles, 2002; Ito & Kitagawa, 2006; Stemmer, Segalowitz, Dywan, Panisset, & Melmed, 2007), which is characterized by nigrostriatal and mesocorticolimbic DA depletion (Agid et al., 1993; Kish, Shannak, & Hornykiewicz, 1988). Of note, these studies are in contrast with a report in which mild to moderate Parkinson’s disease (Holroyd et al., 2002) was not associated with attenuated ERN amplitude as compared to controls.

While there is evidence for involvement of striatal DA in feedback processing, no genetic studies have directly investigated the role of striatal DA in ERN or FRN generation.

A candidate for investigating this hypothesis is the dopamine transporter (DAT). The DAT is much more abundant in the striatum, where it is the major source of DA reuptake, than in the prefrontal cortex (Jaber, Jones, Giros, & Caron, 1997; Wayment, Schenk, & Sorg, 2001). The gene coding for the DAT contains a polymorphism (DAT1) that determines transporter availability (Fuke et al., 2001; Mill, Asherson, Browes, D’Souza, & Craig, 2002; VanNess, Owens, & Kilts, 2005), and therefore striatal DA reuptake. The primary aim of this study was to examine the roles of both striatal (DAT) and prefrontal (COMT) dopaminergic genetics in feedback processing. In contrast to the FRN, where substantial evidence about its general dopaminergic locus is provided but questions remain regarding striatal versus prefrontal modulations, to date and to the best of the authors’ knowledge, no studies have investigated the neuropharmacological locus of the feedback P3.

At the behavioral level, DA genetics have been associated with increased willingness to take financial risks (Kuhnen & Chiao, 2009), pathological gambling (Ibáñez, Blanco, Perez de Castro, Fernandez-Piqueras, & Sáiz-Ruiz, 2003), novelty seeking (Ebstein, Benjamin, & Belmaker, 2000), extraversion (Reuter & Hennig, 2005), impulsivity disorders such as ADHD (Franke et al., 2010), and proneness to addiction disorders (Kreek, Nielsen, Butelman, & LaForge, 2005). However, experimental paradigms that have shown direct involvement of dopaminergic genes in risk taking or reward and punishment sensitivity have been sparse.

In addition to dopamine, previous research (Lesch et al., 1996) has suggested that serotonin is involved in risk and reward processing, which therefore could influence both feedback-related brain activity and risk taking. A candidate gene for testing this hypothesis is the serotonin transporter gene, which comprises a 44-base pair insertion-or-deletion polymorphism (5HTTLPR) generating either a long or a short allele (Heils et al., 1996; Lesch, et al., 1996). Previously, the presence of the short allele has shown to be related to self-reported harm avoidance and neuroticism (Munafò et al., 2009), which are conceptually linked to the risk taking and punishment sensitivity, respectively, and manifest in FRN activity. To date, only two studies have shown direct modulations of 5HTTLPR on neurophysiological error processing in the brain. In adults, it was shown that short-allele carriers (low reuptake, high extracellular 5HT levels) had a larger response-related ERN than did long-allele homozygotes (Fallgatter et al., 2004). In line with this finding, another recent study (Althaus et al., 2009) reported increased ERN amplitudes and less habituation to negative feedback in children carrying the short allele. Congruently, reduced financial risk taking for solely short-allele carriers had been reported earlier (Kuhnen & Chiao, 2009).

Taken together, the current evidence about the role of monoaminergic genes in feedback processing is modest at best. Therefore, the present study aimed to clarify the effects of genetic polymorphisms in striatal (DAT1) and prefrontal (COMT val158met) areas on the neurophysiological response to feedback and risk taking. Furthermore, we aimed to test whether prior reports regarding 5HTTLPR and error processing (ERN) can be translated into feedback processing (FRN), while investigating for the first time the neurogenetics of the feedback P3. Given this rationale, we used a well-established gambling paradigm (Gehring & Willoughby, 2002) while EEG was recorded to investigate the influence of the described polymorphisms (DAT1, COMT val158met, and 5HTTLPR) on both risk-taking behavior/punishment and reward sensitivity and neurophysiological activation following monetary gains and losses.

## Method

### Subjects

A group of 60 healthy female subjects (mean age = 20.87, *SD* = 1.98) was recruited via advertisements at the faculty of Social Sciences at Utrecht University, the Netherlands. Subjects were only included if they were free of medication, drug abuse, and serious medical or neurological injuries that could have confounded the study.

All subjects signed an informed consent form written in accordance with the guidelines of the Ethics Committee of the University Medical Center Utrecht. All further procedures were in compliance with the guidelines of the Review Board for Scientific Research in Humans of the Utrecht University Faculty of Social Sciences.

### DNA extraction and genotyping

DNA was harvested by collecting buccal swabs and was frozen immediately at –40 °C for later genotyping. Genomic DNA was extracted and purified using a QIAamp DNA Mini Kit (Qiagen, Hilden, Germany).

The DAT1 polymorphism was amplified using polymerase-chain reaction (PCR) on a thermal cycler with an initial denaturation at 95 °C for 5 min, followed by 40 cycles of 30 s at 95 °C, 45 s at 60 °C, and 60 s at 72 °C, plus a final elongation of 7 min at 72 °C. Amplification reactions were performed in a total volume of 20 μl, containing approximately 2 μl of genomic template, 10 μl of REDExtract-N-Amp PCR ReadyMix (Sigma-Aldrich, USA) that included Taq polymerase and dNTPs, 0.5 μM of each primer, and 8 μl H_2_O. The primers used in the PCR were as follows: forward primer, 5'-TGT GGT GTA GGG AAC GGC CTG AG-3', and reverse primer, 5'-CTT CCT GGA GGT CAC GGC TCA AGG-3'. Products were electrophoresed in 2 % agarose dissolved in 0.5 TBE buffer, stained with ethidium bromide (0.5 μg/μl), and visualized under ultraviolet (UV) light.

Subjects were further classified on the basis of the presence or absence of the nine-repeat allele. The DAT contains a variable number of tandem repeat (VNTR) polymorphisms in the 3' untranslated region on chromosome 5p15 (Sano, Kondoh, Kakimoto, & Kondo, 1993; Vandenbergh et al., 1992) with a length of 40 base pairs, and it mostly occurs with nine or ten repeats. Whereas in-vivo binding studies regarding DAT1 revealed rather contradictory results (Heinz et al., 2000; van Dyck et al., 2005), in-vitro studies have coherently suggested that the nine-repeat allele is associated with decreased DAT availability and higher DA levels (Fuke et al., 2001; Mill et al., 2002; VanNess et al., 2005). The genotype frequencies were 9/9 (*n* = 3), 9/10 (*n* = 20), and 10/10 (*n* = 37). Subjects were grouped as either nine-repeat-allele (9R) carriers (*n* = 23, 38.3 %) or homozygotes of the ten-repeat allele (10/10, *n* = 37, 61.7 %), as is commonly done with the *SLC6A3* DAT1 polymorphism (Congdon, Lesch, & Canli, 2008; Fuke et al., 2001; Hünnerkopf, Strobel, Gutknecht, Brocke, & Lesch, 2007; Mill et al., 2002). This distribution is in accordance with earlier studies done with respect to the *SLC6A3* DAT1 polymorphism in Caucasians (Hünnerkopf et al., 2007).

The COMT val158met polymorphism (rs4680) was genotyped using a Taqman Drug metabolism genotyping assay (ASSAY ID: C__25746809_50; Applied Biosystems, Foster City, CA). The subjects were classified according to an endpoint analysis performed on an ABI Prism 7000 as either met/met (*n* = 16), val/met (*n* = 33), or val/val carriers (*n* = 8). Subsequently, subjects were grouped as val-allele carriers (*n* = 41, 71.9 %) or met/met homozygotes (*n* = 16, 28.1%) to ascertain sufficient statistical power.

5HTTLPR genotyping was based on a published protocol (Lonsdorf et al., 2009) with some adjustments (see Londorf’s 2011 erratum). The primers used in this procedure were as follows: forward primer, 5*'*-GGCGTTGCCGCTCTGAATGC-3', and backward primer, 5'-GAGGGACTGAGCTGGACAACCAC-3'. Products were electrophoresced in 2 % agarose dissolved in 0.5 TBE buffer, stained with ethidium bromide (0.5 μg/μl), and visualized under UV light. The short allele has reduced transcriptional efficiency in the promoter region and is therefore associated with lower transporter reuptake and, presumably, higher brain serotonin levels. The genotype frequencies were s/s (*n* = 12), s/l (*n* = 25), and l/l (*n* = 17). To assure sufficient statistical power, subjects were grouped as either short-allele carriers (*n* = 37, 68.5%) or carriers of two long alleles (l/l; *n* = 17, 31.5 %). Genotyping procedures could not be completed in three subjects for the COMT polymorphism and six subjects for the 5HTTLPR polymorphism.

### Gambling task

A gambling task (Gehring & Willoughby, 2002) was used to assess risk taking and prefrontal brain response to feedback. The task comprised five blocks with 32 trials each. Subjects were presented with two squares with monetary rewards of 5c or 25c (in this case, 5 or 25 euro cents) displayed inside, leading to four possible stimuli: 5c–25c, 25c–5c, 5c–5c, and 25c–25c. The subjects could choose one of the two squares by pressing the corresponding button, and at 1,000 ms postchoice, each square turned either red or green, indicating a gain (green) or a loss (red). The intertrial interval was 1,500 ms. After being familiarized with the task in a practice block, subjects started with a balance of €2.50 and were encouraged to gain as much money as possible. Importantly, the subjects were instructed that there was “no optimal strategy to learn.” The mean expected monetary outcome was zero, in order to avoid potential confounding influences of different gain or loss probabilities. A one-sample *t* test for balance after each block revealed no significant deviance from €0 in each block, on average (all *p* values > .2). It should be noted that adding the total amount of money won during the task as a covariate in all tests on risk-taking behavior and EEG amplitudes did not result in changes in the findings from significant to nonsignificant, or vice versa. Subjects were informed of their actual gains and losses at the end of each block, and the earned money was paid to the subjects at the end of the task. Risk-taking behavior during gambling was assessed by computing three different parameters: the percentage of risky choices (choosing 25c over 5c) during the task, the percentage of risky choices following loss trials, and the percentage of risky choices following gain trials. Whereas the first parameter allowed for investigating risk-taking tendencies in general, computing choices specifically following loss and gain trials was done to assess punishment and reward sensitivity.

### EEG measurements

The electroencephalogram (EEG) was recorded at 30 scalp sites corresponding to midline (Fz, Fcz, Cz, Cpz, Pz, Oz), frontal (Fp1, Fp2, F3, F4, F7, F8, FT7, FT8), central (C3, C4, FC3, FC4), temporal (T7, T8, TP7, TP8), parietal (P5, P6, CP3, CP4), and occipital (O1, O2, Oz) locations according to the international 10–10 system, using an electrode cap (QuikCap, Neurosoft, El Paso, TX) with tin electrodes. The right mastoid was used as a reference for all recordings. A horizontal electrooculogram (HEOG) was recorded with two electrodes from the outer canthi of each eye, and a vertical electrooculogram (VEOG) was recorded with two electrodes placed infra- and supra-orbital of the left eye. The ground electrode was placed at AFz, and impedance was kept below 10 kΩ for each electrode. All signals were amplified by two Synamps amplifiers with an online low-pass filter at 50 Hz and a high-pass filter at 0.05 Hz. Data were recorded with a sampling rate of 500 Hz with Neuroscan software version 4.2 (Neurosoft, El Paso, TX). Offline, the data were processed using Brain Vision Analyzer software, version 1.05 (Brain Products, Gilching, Germany). The signal, down-sampled to 256 Hz, was filtered with a low-cutoff filter at 0.15 Hz and a high-cutoff filter at 30 Hz, both with a slope at 24 dB/oct. Ocular artifacts were corrected using the method proposed by Gratton, Coles, and Donchin (1983).

### ERPs

After the EEG corrections described above, trials were segmented, grouped (gain and loss), averaged, and baseline-corrected relative to a 100-ms prestimulus interval. Segmented trials with artifacts were removed semi-automatically if the signal either exceeded a 100-μV within-segment absolute difference criterion or showed less than 0.5 μV activity within 100 ms.

As the FRN is superimposed on the feedback positivity, we chose a peak-to-peak approach to disentangle the feedback-related negativity and positivity. This approach has successfully been applied in various other studies (Beste et al., 2010; Beste et al., 2008; Frank et al., 2007; Krämer et al., 2007) and minimizes possible distortions of the FRN component by the positivity on which it is superimposed. Therefore, FRN amplitudes were quantified as the average activity within time windows of ±10 ms around the peak latency of the preceding positivity (190–210 ms) minus the subsequent negativity (240–260 ms). Larger values, hence, indicate greater negativity. This computation was applied to loss trials and gain trials separately. In addition, the loss-FRN-minus-gain-FRN difference was computed. Feedback P3 amplitudes for gains and losses were computed as the average activity between 280 and 360 ms at frontal (Fz) and parietal (Pz) sites. Two subjects were excluded due to large amounts of artifacts (more than 25 % of trials rejected, whereas the mean percentage of artifact trials was <5 %) at the target electrode (Fz). This resulted in valid ERP data from *n* = 58 out of *n* = 60 subjects (96.67 %).

### Statistics

Analyses of variance (ANOVAs) and repeated measures ANOVAs were conducted whenever appropriate. The Greenhouse–Geisser correction was used whenever sphericity could not be assumed. Interaction effects between genes were not computed, due to the fact that the sample size did not yield sufficient statistical power. An alpha of .05 was used for all tests (two-tailed).

## Results

### Genetic distribution

All genotypes were in Hardy–Weinberg equilibrium (*p >* .2) and linkage equilibrium (all *p* values > .14). The genotype frequencies and descriptive statistics are shown in Table 1.

**Table 1:** Descriptive information and statistics about risk-taking behavior and electrophysiological activity during the gambling paradigm, dependent on genetic polymorphisms in DAT1, COMT val158met, and 5HTTLPR

		DAT1		COMT val158met		5HTTLPR	
		9R-Carriers	10/10	met/met	val-Carriers	s-Carriers	1/1
	N	23	37	16	41	37	17
Risk Taking							
% Risky Choices	M ± SD	62 ± 18	53 ± 21	59 ± 16	55 ± 21	53 ± 22	62 ± 12
	F	2.88		1.24		2.63	
	p	.095		.52		.11	
	η^2^	.047		–		–	
% Risky Choices Post Loss	M ± SD	64 ± 19	53 ± 22	60 ± 17	55 ± 22	54 ± 23	59 ± 17
	t	1.89		0.82		0.74	
	p	.064		.41		.47	
	d	.39		–		–	
% Risky Choices Post Gain	M ± SD	60 ± 20	54 ± 21	57 ± 19	56 ± 21	52 ± 22	64 ± 11
	t	1.10		0.31		4.82	
	p	.28		.76		.008	
	d	–		–		.69	

FRN							
Loss – Gain FRN (μV)	M ± SD	3.80 ± 2.49	2.20 ± 2.27	3.09 ± 3.21	3.09 ± 3.21	3.36 ± 2.64	1.84 ± 1.85
	F	6.31		0.11		4.51	
	p	.015		.74		.039	
	η^2^	.101		–		.083	
Loss FRN (μV)	M ± SD	4.04 ± 2.28	2.49 ± 3.46	2.30 ± 2.40	3.19 ± 3.21	3.52 ± 2.93	1.50 ± 2.78
	t	2.05		0.99		2.37	
	p	.046		.33		.022	
	d	.615		–		.707	
Gain FRN (μV)	M ± SD	0.24 ± 2.73	0.29 ± 3.51	–0.79 ± 2.93	0.35 ± 3.01	0.16 ± 3.13	–0.35 ± 2.83
	t	0.07		1.28		0.56	
	p	.95		.21		.58	
	d	–		–		–	

Feedback P3							
Loss – Gain P3 (μV)	M ± SD	–1.25 ± 2.30	–1.32 ± 2.84	–1.65 ± 2.62	–1.01 ± 2.63	–1.30 ± 2.77	–1.11 ± 2.46
	F	0.01		0.82		0.06	
	p	.92		.42		.81	
	η^2^	–		–		–	
Loss P3 (μV)	M ± SD	12.13 ± 6.30	10.62 ± 4.37	13.43 ± 5.99	10.26 ± 4.61	11.12 ± 4.78	11.73 ± 6.20
	t	1.07		2.11		0.39	
	p	.29		.040		.70	
	d	–		.59		–	
Gain P3 (μV)	M ± SD	13.38 ± 6.75	11.94 ± 4.99	15.08 ± 6.09	11.27 ± 5.20	12.42 ± 5.82	12.84 ± 5.89
	t	0.92		2.34		0.24	
	p	.36		.023		.81	
	d	–		.67		–	

### Behavioral results

There were no significant differences between the amounts of money subjects won during gambling and their DAT1, COMT val158met, and 5HTTLPR genotypes (all *p* values > .3). Risk taking was defined as the average percentage of choosing 25c (high value) over 5c (low value), and was analyzed as a function of winning or losing in the previous trial.

**Fig. 1:**
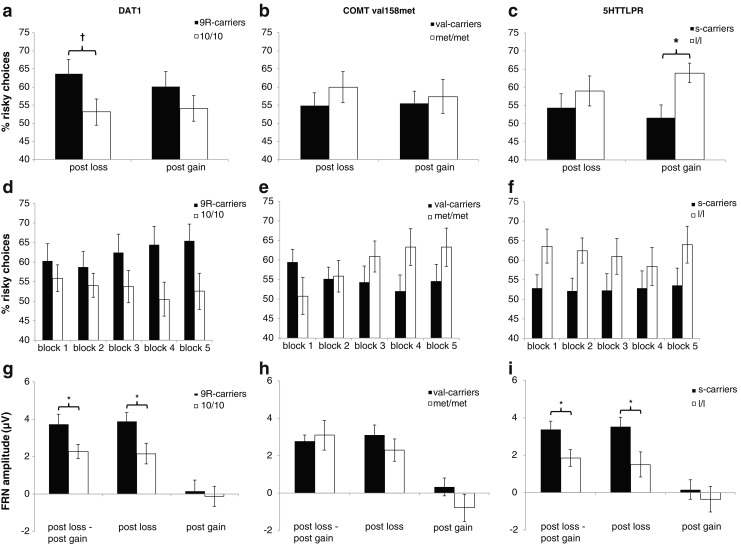
Behavioral and electrophysiological data from the gambling paradigm are depicted dependent on genetic polymorphisms within DAT1 (left), COMT val158met (center), and 5HTTLPR (right). (**a**–**c**) Average percentages of risky choices following losses and gains. (**d**–**f**) Time course of risk-taking behavior, displayed as average percentages of risky choices for the five blocks of the gambling task. (**g**–**i**) Amplitudes of the loss FRN, the gain FRN, and the loss FRN – gain FRN difference are shown. Error bars depict ±1 *SEM*. ^*^ *p* < .05. ^†^ *p* < .10

For the percentage of risky choices following losses, we found a marginally significant effect of DAT1 genotype [*F*(1, 58) = 3.56, *p* = .064, *η* ^2^ = .06], showing a tendency for more risk taking in 9R-carriers of DAT1, but no effect for the 5HTTLPR (*F* < 1) or COMT val158met (*F* < 1) genotype. Following gains, there was a significant effect of 5HTTLPR genotype [*t*(52) = 4.82 *p* = .008, *d* = 0.69] showing reduced risk taking in s-carriers, but no effect for DAT1 [*t*(58) = 1.20, *p* = .28] or COMT (*t* < 1) genotype; see Fig. 1 and Table 1 for an overview of the results.

Furthermore, repeated measure ANOVAs were conducted using Genotype as a between-groups factor and Percentage of Risky Choices in each of the five gambling task blocks as a within-subjects factor. By means of this test, changes in risk-taking behavior throughout the task—for instance, increase or decrease in risk taking—could be assessed. There was no significant difference in risk-taking behavior independent of genotype throughout the task (*F* < 1), meaning that risk taking did not change overall during the five blocks. However, there was a significant effect of COMT genotype [*F*(5.7, 155.16) = 3.18, *p* < .01, *η* ^2^ = .11] and a borderline significant effect of DAT1 genotype [*F*(2.86, 166.02) = 2.57, *p* = .059, *η* ^2^ = .04] on changes in risk-taking behavior during the task. 5HTTLPR genotype was not significantly related to changes in risk-taking behavior throughout the task (*F* < 1). See Fig. 1 for an illustration of these patterns. To ascertain that risk-taking behavior did not confound the ERP results, the average amount of risky choices during gambling was used as a covariate in all of the subsequent EEG analysis. It should be noted that the addition of this covariate in all subsequent tests did not change any results from significant to nonsignificant, or vice versa.

### Electrophysiological results

#### Valence effects (FRN)

**Fig. 2:**
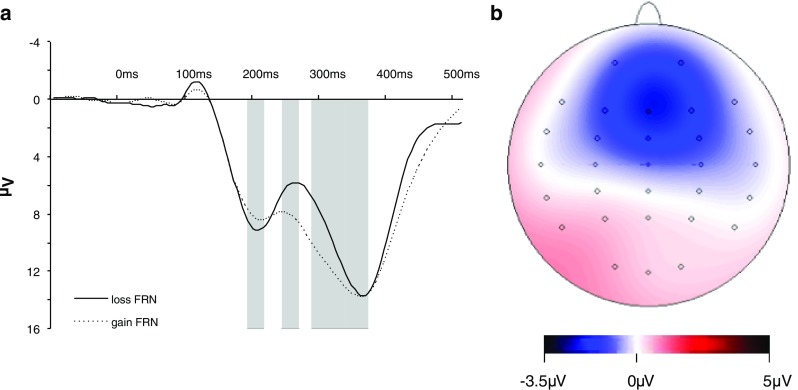
**a** Grand-average waveforms of all subjects (*n* = 58) following gains (dotted line) and losses (solid line) at Fz. A large increase in FRN amplitude is observed following losses. The time windows to compute the FRN comprised the average activity from 190 to 210 ms minus the average activity from 240 to 260 ms at Fz. Feedback P3 amplitudes were computed as the average activity from 280 to 360 ms. These time windows are shown in gray. **b** Scalp distribution of the FRN component derived from the loss FRN – gain FRN difference between 242 and 262 ms. A clear frontocentral distribution is observed

A repeated measures ANOVA with loss FRN versus gain FRN as dependent variables revealed a significant effect of valence [*F*(1, 56) = 75.23, *p* < .001, *η* ^2^ = .57], showing that the peak-to-peak loss FRN was much larger than the peak-to-peak gain FRN (see Fig. 2).

#### Valence × genotype effects (FRN)

**Fig. 3:**
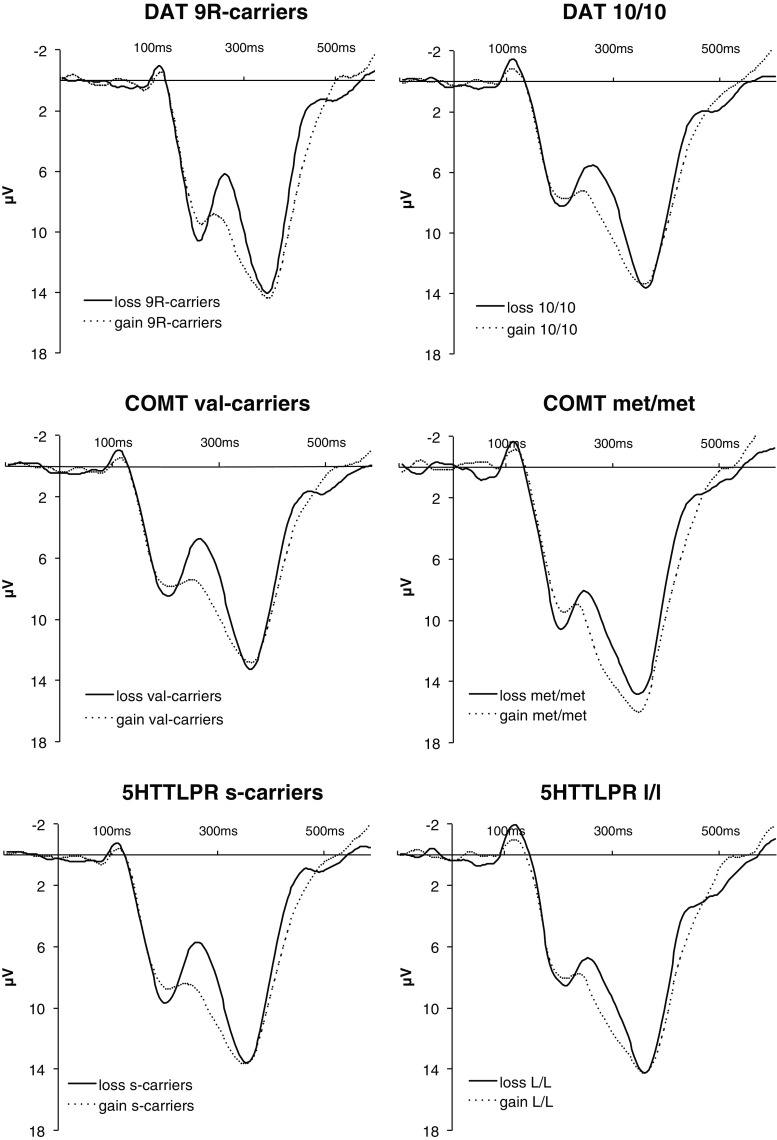
Grand-average waveforms at the midfrontal electrode site (Fz) following gains (dotted lines) and losses (solid lines) during gambling, dependent on DAT1 (top), COMT val158met (center), and 5HTTLPR l/l (bottom) genotypes

**Fig. 4:**
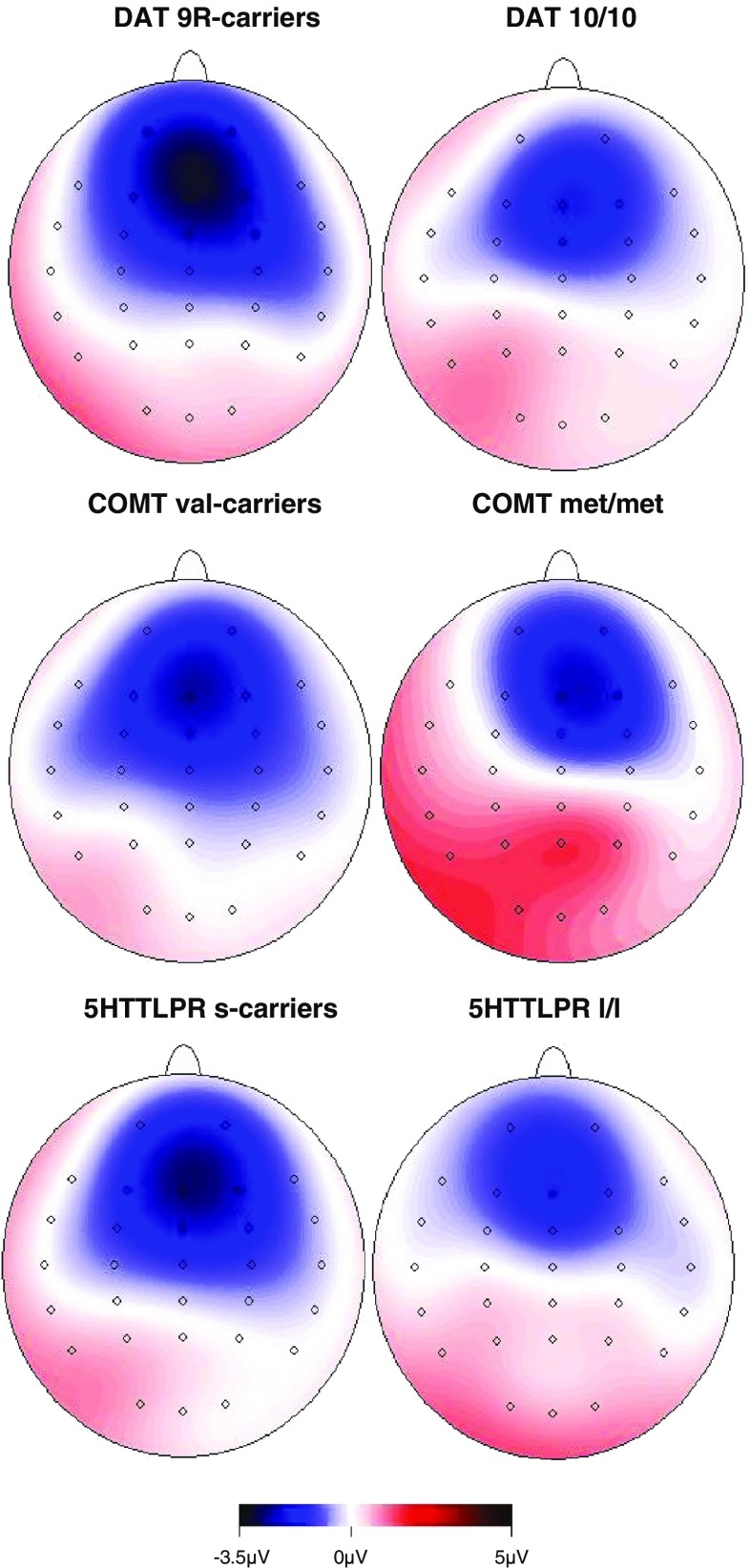
Skull distribution of activity from the loss minus gain difference wave from 240 to 260 ms postfeedback, dependent on DAT1 (top), COMT val158met (center), and 5HTTLPR l/l (bottom) genotypes. Blue color indicates negativity, red color indicates positivity

Then we tested, for each polymorphism, whether this valence effect computed as the loss FRN – gain FRN difference in amplitude was modulated by the genetic polymorphisms under study. There was a significant DAT1 Genotype × Valence interaction [*F*(1, 56) = 6.30, *p* = .015, *η* ^2^ = .10], revealing that 9R-carriers showed a larger difference than did 10/10 homozygotes. In addition, we found a significant 5HTTLPR Genotype × Valence interaction [*F*(1, 50) = 4.51, *p* = .039, *η* ^2^ = .08], revealing that s-carriers showed a larger difference than did l/l carriers. However, there was no COMT Val158met Genotype × Valence interaction (*F* < 1). See Figs. 3 and 4 and Table 1 for an overview of these results. It should be noted that cursory inspection of the FRN amplitudes seems to depict a larger FRN for COMT val-carriers than for met/met homozygotes. This was, however, attributable to a difference in overall amplitude (see the next paragraph) rather than to a valence-dependent difference in peak-to-peak FRN amplitudes.

#### Genotype effects on loss FRN and gain FRN separately

In addition to the previous analyses of the loss FRN – gain FRN difference, we tested whether the genetic polymorphisms under study were specifically related to the gain FRN and/or the loss FRN amplitude. As is shown in Table 1 and Figs. 2 and 3, loss FRN amplitudes were significantly modulated by DAT1 [*t*(57) = 2.05, *p* = .046, *d* = 0.62] and 5HTTLPR [*t*(51) = 2.37, *p* = .02, *d* = 0.71] genotypes, showing larger FRNs for DAT1 9R-carriers and 5HTTLPR s-carriers when compared to the respective other genotypes. In the contrary, there were no modulations by the genetic polymorphisms under study of the gain FRN amplitudes (*p* values > 2).

#### Genotype × feedback P3 effects

In addition, we tested whether the feedback P3 was modulated by the genetic polymorphisms under study. We found no effects of DAT1, COMT val158met, or 5HTTLPR genotype on the feedback P3 loss minus gain difference at frontal (Fz) and parietal (Pz) electrode sites (all *p* values > .4). However, there was a significant effect of COMT val158met on frontal feedback P3 amplitudes (Fz) following both losses [*t*(52) = 2.11, *p* = .04, *d* = 0.59] and gains [*t*(52) = 2.34, *p* = .023, *d* = 0.67; see Fig. 3], showing an increased P3 amplitude for met/met homozygotes as compared to val-allele carriers. In contrast, the DAT1 genotype and 5HTTLPR genotype were unrelated to the frontal feedback P3 amplitude (all *p* values > .2). At the parietal electrode site (Pz), there were no effects of the polymorphisms under study on the feedback P3 (all *p* values *>* .5).

## Discussion

The main aim of the present study was to investigate a possible dissociation between prefrontal and striatal dopaminergic genetics in human feedback processing and risk taking. Sixty healthy female subjects completed a gambling paradigm (Gehring & Willoughby, 2002) to measure individual risk taking while the feedback-related negativity (FRN) and feedback P3 were recorded using EEG. Subsequently, modulations by the genetic polymorphisms under study (DAT1, COMT val158met, and 5HTTLPR) of neurophysiological feedback response and punishment and reward sensitivity were investigated.

As a candidate gene coding for striatal DA levels, we investigated the dopamine transporter (DAT1) polymorphism. As mentioned earlier, DAT expression is very high in the striatum but plays only a minor role in prefrontal DA reuptake (Sesack, Hawrylak, Guido, & Levey, 1998; Wayment et al., 2001). The presence of the nine-repeat allele of DAT1 has been associated with decreased transporter expression as compared to the ten-repeat allele, presumably leading to higher tonic DA levels in the striatum (Bannon, Michelhaugh, Wang, & Sacchetti, 2001; Fuke et al., 2001; Mill et al., 2002; van Dyck et al., 2005; VanNess et al., 2005). In this study, presence of the nine-repeat allele of DAT1 was associated with larger FRN amplitudes, as compared to 10/10 homozygotes. Follow-up analyses revealed that this effect on FRN amplitudes was related to increased prefrontal activation following losses specifically, but not following gains. In addition to the neurophysiological effect, DAT1 9R-carriers tended to display increased risk-taking tendencies during gambling, which were mainly present following losses (*p =* .064).

COMT val158met, mainly related to prefrontal rather than striatal DA levels, was not related to FRN amplitudes in the present study. However, met/met homozygotes displayed increased prefrontal feedback P3 amplitudes following both losses and gains as compared to val-carriers. In addition, the met/met group showed a progressive increase in risk taking with longer task duration but no effects on risk taking following losses or gains. This progressive increase in risk taking toward the end of the task might be explained by the knowledge that the overall reward obtained during the experiment will not end up to be negative, which might have invited subjects to make more “risky” choices toward the end.

As stated earlier, a key question of this study was whether candidate genes modulating DA in mainly the striatum or the prefrontal cortex are related to the neurophysiological response to feedback and risk taking. Holroyd and Coles’s (2002) reinforcement learning theory states that reward prediction errors lead to transient pauses of DA release in the anterior cingulate, which in turn generates a FRN or an ERN. Following their account, a genetic polymorphism in the main enzyme determining prefrontal DA levels (COMT) would be expected to modulate the neurophysiological response to prediction error. However, this was not the case in the present study, as COMT was unrelated to FRN amplitude.

Other authors have noted limitations to reinforcement learning theory (Jocham & Ullsperger, 2009; Ullsperger, 2010), mostly because dopaminergic neurotransmission does not seem to possess the temporal accuracy to encode an ERN or FRN. Alternative frameworks have been proposed (Frank et al., 2007; Frank, Doll, Oas-Terpstra, & Moreno, 2009; Jocham & Ullsperger, 2009; Lapish, Kroener, Durstewitz, Lavin, & Seamans, 2007; Ullsperger, 2010), in which other, faster neurotransmitters, such as glutamate and GABA, encode the prediction error. DA, in these models, still plays an important role by setting the background of excitability in the cortex and striatum. According to these frameworks, one would expect striatal rather than prefrontal DA as a determinant of neurophysiological feedback response. Our findings, a significant effect of DAT1 but not of COMT val158met on FRN amplitude, concur with this view. Converging evidence for striatal involvement in the generation of the ERN has been provided by studies investigating Parkinson’s disease (see, e.g., Falkenstein et al., 2001; Ito & Kitagawa, 2006; Stemmer et al., 2007), which is characterized by the depletion of striatal DA (Agid et al., 1993; Kish et al., 1988). Of note, these studies are in contrast with a report in which mild to moderate Parkinson’s disease (Holroyd et al., 2002) was not associated with attenuated ERN amplitudes as compared to controls. While there is evidence for the involvement of striatal DA in feedback processing, no genetic studies have directly investigated the role of striatal DA in ERN or FRN generation.

However, it should be noted that both reinforcement learning theory and alternative frameworks proposed more recently (Frank et al., 2007; Jocham & Ullsperger, 2009; Ullsperger, 2010) do not necessarily rule out each other. As such, Holroyd and Coles’s (2002) model portrays that DA-mediated temporal difference signals modulate both the basal ganglia (as an adaptive critic) and the ACC (as the control filter). The basal ganglia influence the motor controllers, whose striatal DA-modulated input is subsequently fed into the ACC. In addition, Frank et al. (2007) argued that striatal and prefrontal mechanisms might work in tandem. Whereas the subcortical DA system could initially train the medial prefrontal cortex to recognize errors, the medial prefrontal cortex itself might drive DA dips for maladaptive decisions after initial learning has taken place.

In addition to our electrophysiological results, risk-taking patterns also showed differentiation of striatal versus prefrontal dopaminergic genetics in the present study. As such, DAT1 genotype showed a trend-level association with risk taking directly after a loss was encountered, suggesting trial-by-trial behavioral adjustments. In contrast, COMT val158met genotype was associated with a propensity to increase risk taking over the course of the gambling task, possibly indicating increased sensation or novelty seeking (Reuter & Hennig, 2005).

We furthermore found that feedback P3 amplitude was specifically related to the mainly prefrontal dopaminergic polymorphism (COMT val158met), but not to the mainly striatal polymorphism (DAT1) under study. It was striking to see that especially the frontal (Fz), but not the parietal (Pz), portion of the feedback P3 was increased in met/met carriers, who are thought to have higher prefrontal DA levels. Congruently, it has been reported earlier that the error positivity, an ERP closely related to the feedback P3, is modulated by COMT. Frank et al. (2007) found an increased error positivity for met/met carriers but no COMT effects on ERN amplitudes, concurring with our results. It has furthermore been argued (Yeung & Sanfey, 2004) that the P3 component in gambling tasks reflects the reward amount rather than the outcome valence. This idea is also supported by the present results, as COMT val158met affected feedback-P3 amplitude following both losses and gains, with no effects on the loss-minus-gain feedback P3 difference. The notion that prefrontal DA signaling is involved in the generation of frontal P3 components is not new and has been proposed in other domains, such as novelty processing (Marco-Pallarés et al., 2010) or performance monitoring (Càmara et al., 2010; Krämer et al., 2007).

In sum, the dopaminergic findings of the present study show a pattern of results that suggests that striatal DA is an important factor in FRN generation and risk-taking tendencies, while prefrontal DA modulates the feedback P3 and behavioral adjustments that evolve during the task. Even though these findings are in agreement with several other reports (Frank et al., 2007; Krämer et al., 2007), it should be noted that a recent study reported contrasting results regarding COMT val158met (Marco-Pallarés et al., 2009). In that previous study, COMT val158met genotype *did* significantly modulate FRN amplitudes. Several methodological differences could have contributed to this discrepancy. First of all, the FRN is superimposed on the feedback P3, which makes it difficult to dissociate these ERPs. We chose to compute peak-to-peak differences between the preceding positive peak and the actual negativity in order to prevent P3 differences from being incorporated into the FRN. Similar approaches have been used by other authors (Beste et al., 2010; Beste et al., 2008; Frank et al., 2007; Krämer et al., 2007). In contrast, Marco-Pallarés et al. (2009) computed a loss-minus-gain difference wave (250–300 ms) in order to quantify FRN amplitudes, similar to the technique of Holroyd et al. (2006). Furthermore, we only used female subjects, while the previous sample included both sexes. As has been shown before, COMT effects might be sex-dependent, as transcription (Jiang, Xie, Ramsden, & Ho, 2003; Xie, Ho, & Ramsden, 1999), basal transporter levels (Guo, Wu, Liu, Yang, & Chen, 2004; Tunbridge, Burnet, Sodhi, & Harrison, 2004), and the mechanisms by which COMT acts are sexually dimorphic (Gogos et al., 1998). A final difference is that Marco-Pallarés et al.’s (2009) analysis was based on a subdivision of the subjects on the basis of homozygosity of the DRD4 and COMT genotypes, whereas heterozygotes as well were used in the present study.

In addition to the involvement of dopaminergic genes, we investigated a possible serotonergic contribution toward risk taking and the FRN by genotyping subjects for a common polymorphism in the serotonin transporter gene (5HTTLPR). FRN amplitudes were increased for short-allele carriers of 5HTTLPR as compared to long-allele homozygotes. This concurs with previous reports of serotonergic modulations of neurophysiological performance-monitoring measures (Althaus et al., 2009; Beste et al., 2010; Fallgatter et al., 2004; Olvet, Hatchwell, & Hajcak, 2010). To the authors’ knowledge, the present study is the first to show a 5HTTLPR-dependent modulation of the feedback-related negativity induced by monetary gains and losses. Serotonin has been related to motivational valence and error signaling, and it has been proposed that serotonergic signaling interacts with dopamine signaling in the processing of reward and punishment (Cools, Roberts, & Robbins, 2008). Furthermore, others have argued that serotonin modulates the motivational valence of feedback and cortical excitability (Jocham & Ullsperger, 2009). Coherent with this hypothesis, short-allele carriers displayed less risk taking during gambling, on average, and in particular following gain trials. This might point toward a rather cautious gambling strategy in 5HTTLPR s-carriers, which is in line with the fact that short-allele presence is associated with harm avoidance and neuroticism (Munafò et al., 2009). Taken together, research directly linking serotonin to these processes is rather scarce. Here we have presented for the first time evidence that serotonin is involved in feedback processing. In order to understand the role of serotonin in more detail, further studies are warranted. In that respect, the investigation of 5HTTLPR seems to be a promising avenue.

Furthermore, it should be pointed out that the serotonergic (as indexed by 5HTTLPR genotype) modulation of risk-taking behavior is dissociated from the dopaminergic modulation (as indexed by a trend-level effect of DAT1 genotype). While DAT1 9R-carriers showed a tendency toward more risk taking following losses, 5HTTLPR short-allele carriers displayed significantly decreased risk taking following gains. Neuropharmacologically, this could be explained by more striatal dopamine (9R DAT1) leading to more risky behavior despite the just-experienced loss, whereas more serotonin would translate to less risky behavior despite the just-experienced gain, which would be in accordance with the current literature on the role of striatal dopamine in reward approach behavior versus the role of serotonin in loss avoidance behavior (see Boureau & Dayan, 2011, for a review of this topic).

When interpreting the findings of the present study, several methodological limitations should be kept in mind. First of all, we investigated females only, due to logistical reasons. This obviously limits the generalization of our conclusions to females. In addition, gonadal steroids are known to interact with the dopaminergic system, depending on the phase of the menstrual cycle (Becker & Cha, 1989; Creutz & Kritzer, 2004), which could have been a confounding factor. Replication of our findings in male populations is therefore required. Second, our sample size (*n* = 60) did not allow for a meaningful gene × gene interaction analysis. Future studies should therefore incorporate larger samples in order to test for possible interactions. Third, the candidate gene approach does not preclude the possibility that other genes may also be involved. However, it does provide a useful tool for theory-driven dissociations, as was done in the present study. Fourth, we did not perform tri-allelic genotyping of the 5HTTLPR in the present study, meaning that rs25531 within 5HTTLPR was not taken into account. Rs25531 allows for a subdivision of the 5HTTLPR genotype into A-allele or G-allele carriers (Kraft, Slager, McGrath, & Hamilton, 2005), which could potentially lead to 5HTT genotype groups that would correspond more accurately to 5HTT expression levels (Hu et al., 2006). Future studies should therefore take rs25531 into account when investigating 5HTTLPR. Fifth, we did not investigate neurophysiological responses based on the previous trial outcome (e.g., losses following gains) in the present study, as further dissection of the ERP data would have resulted in too low an amount of trials per condition to allow for valid estimates.

In conclusion, we have here shown a genetic dissociation between striatal (DAT1) versus prefrontal (COMT val158met) dopamine at different stages of neurophysiological feedback processing and risk taking. In addition, we have provided novel evidence regarding the involvement of serotonin (5HTTLPR) in the generation of the FRN and risk taking.
